# Metachronous lesions in the orbit, retroperitoneum, and pleura of mucosa‐associated lymphoid tissue lymphoma: A case report

**DOI:** 10.1002/cnr2.1689

**Published:** 2022-07-28

**Authors:** Ayako Shiono, Hisao Imai, Tsugumi Satou, Ryo Taguchi, Naoki Takahashi, Ryuichi Azuma, Ou Yamaguchi, Kosuke Hashimoto, Erika Naito, Hidetoshi Iemura, Yu Miura, Atsuto Mouri, Kyoichi Kaira, Kunihiko Kobayashi, Hiroshi Kagamu

**Affiliations:** ^1^ Department of Respiratory Medicine, Comprehensive Cancer Center International Medical Center, Saitama Medical University Hidaka Japan; ^2^ Department of Pathology, Comprehensive Cancer Center International Medical Center, Saitama Medical University Hidaka Japan; ^3^ Department of Thoracic Surgery Comprehensive Cancer Center, International Medical Center, Saitama Medical University Hidaka Japan; ^4^ Department of Hematopoietic Tumor Comprehensive Cancer Center, International Medical Center, Saitama Medical University Hidaka Japan; ^5^ Department of Plastic Surgery National Defense Medical College Tokorozawa Japan

**Keywords:** metachronous lesions, mucosa‐associated lymphoid tissue lymphoma, orbit, pleural effusion, retroperitoneum

## Abstract

**Background:**

Mucosa associated lymphoid tissue (MALT) lymphoma of the orbit is rare, often indolent, but can recur, and spread to extra‐nodal sites. Pleural and retroperitoneum recurrences of MALT lymphoma are rare.

**Case:**

A 65‐year‐old man was referred to our hospital due to right pleural effusion and difficulty in breathing. He had a medical history of having undergone surgery for MALT lymphoma of the left orbit. A chest computed tomography (CT) scan showed right pleural thickness, pleural effusion, and a retroperitoneal mass, spreading from the muscular layer to the subcutaneous layer. The thickened pleural lesion was surgically biopsied and diagnosed as a recurrence of MALT lymphoma.

**Conclusion:**

Pleural effusion should be carefully examined and monitored for the possibility of recurrence in MALT lymphoma patients.

## 1 INTRODUCTION

Extranodal marginal zone lymphoma of mucosa‐associated lymphoid tissue (MALT lymphoma) is defined as an extranodal low‐grade B‐cell lymphoma, accounting for 7% to 8% of non‐Hodgkin's lymphoma (NHL) cases.^1^, ^2^ MALT lymphoma of the orbit is rare, accounting for less than 1% of NHL cases.^3^ While it exhibits an indolent disease course, MALT lymphoma has reportedly recurred and spread to extranodal sites, requiring long‐term monitoring of progress. Reportedly, 27% of patients with ocular adnexal lymphomas experienced recurrence after initial treatment.^4^ However, pleural and retroperitoneal invasion of MALT lymphoma are rare.^5^, ^6^ We report a case of metachronous recurrence in the right pleura, retroperitoneum, and orbits after treatment of primary MALT lymphoma of the orbit.

## 2 CASE REPORT

**FIGURE 1:**
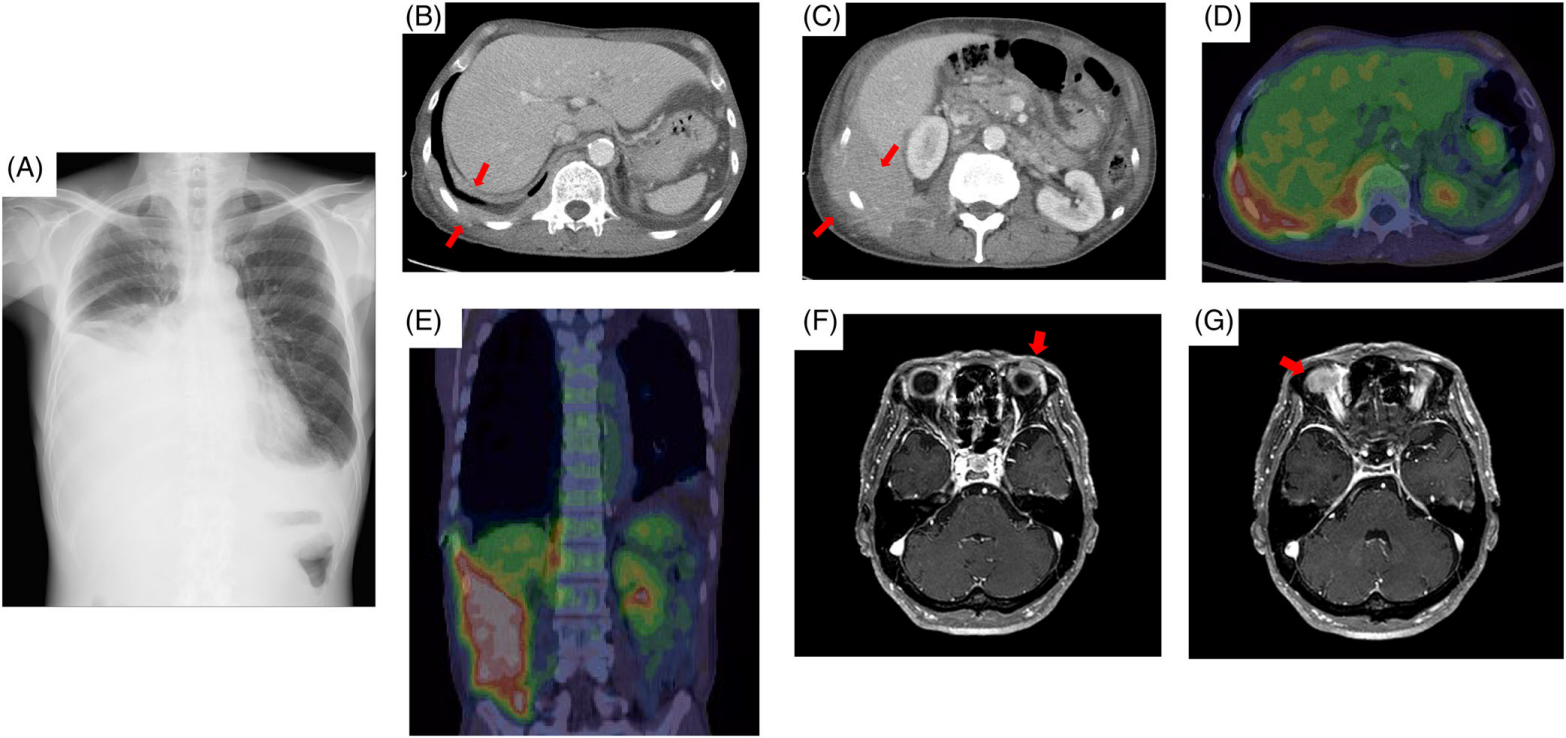
Diagnostic imaging of the patient (A) Chest radiograph shows moderate pleural effusion of the right thoracic cavity. (B, C) Chest computed tomography (CT) scan revealed a right pleural thickening and mass in the retroperitoneum, which spread from the muscular layer to the subcutaneous layer. The image was scanned after thoracic drainage was completed. (D, E) Positron emission tomography (PET) imaging demonstrated increased accumulation of 2‐deoxy‐2‐[18F] fluoro‐d‐glucose (^18^F‐FDG) in the right pleural thickening and the mass of the right retroperitoneum with maximal standardized uptake value (SUV_max_) of 10.51. (F, G) Thickening of the bilateral bulbar conjunctiva on magnetic resonance imaging (MRI) (arrow)

**FIGURE 2:**
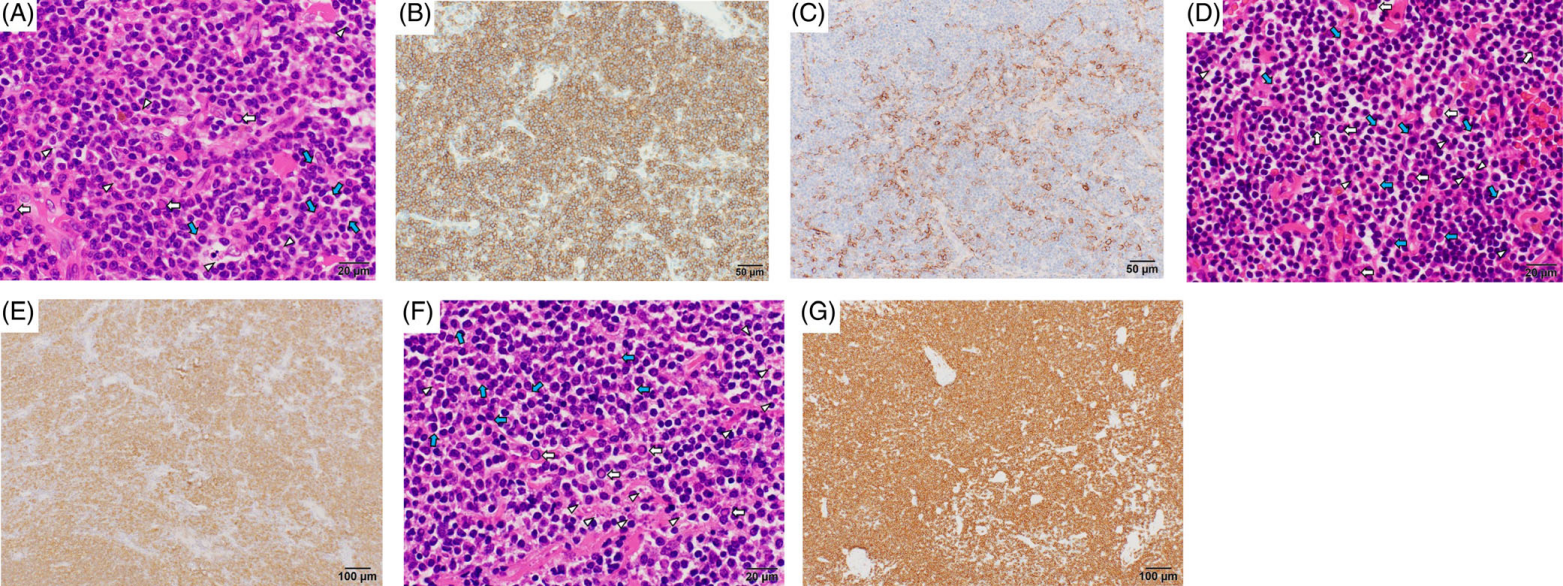
Histological and immunohistochemical findings. (A) Lymphoid cells were observed in the right pleura, with small to medium nuclei presenting as mildly constricted. There are Dutcher bodies in the cells (white arrows) and lymphoglandular bodies (arrowheads). The plasma cells are focally scattered and partially aggregated (blue arrows) (stain, hematoxylin and eosin stain, magnification, x600). Immunohistochemical analysis showed that the lymphoid cells were positive for (B) CD20, and the plasma cells were positive for (C) CD138 (magnification, x200). (D) Left orbit lymphoma in 2009 and (F) Right orbit lymphoma in 2015 showed a diffuse increase in small to medium‐sized lymphocytes (hematoxylin and eosin stain, magnification, x600). These cells expanded the interfollicular space and underwent partial plasmacytoid differentiation, and there were also plasma cells (blue arrows). Some cells had Dutcher bodies (white arrows). Lymphoglandular bodies were also noted (arrowheads). Immunohistochemical analysis of (E) left orbit lymphoma in 2009 and (G) right orbit lymphoma in 2015 showed that the lymphoid cells were positive for CD20 (magnification, x100)

A 65‐year‐old man with no history of chronic pleural inflammation, such as tuberculosis, was referred to our hospital due to right pleural effusion with difficulty in breathing. His right lung breath sounds were diminished. He had a medical history of surgery for MALT lymphoma of the left orbit at a previous hospital in 2009. Six years after the surgery, the patient experienced MALT lymphoma recurrence in the right orbit. Thus, he underwent surgical resection for recurrent lymphoma. He was given no other treatment than surgery in 2009 and 2015. Meanwhile, a retroperitoneal mass measuring 32 x 7 mm in size was detected on computed tomography (CT) in 2015. He discontinued his visits to the initial hospital and was not followed up. Eleven years after the initial diagnosis of MALT lymphoma, he visited our hospital because of dyspnea and massive pleural effusion (Figure 1A). Laboratory examination showed hyperproteinemia (9.5 g/dl), hypoalbuminemia (2.0 g/dl), and high serum IgM levels (6335 mg/dl; normal range 33–183 mg/dl). In addition, immunoelectrophoretic examination revealed monoclonal IgM with κ light chain restriction in both the serum and right pleural effusion. IgM measurement was not performed at the previous hospital, so its accurate determination was not possible. The histological findings of the right pleural fluid suggested a lymphoma. A chest CT scan showed right pleural thickness, right pleural effusion (Figure 1B), and a right retroperitoneal mass, spreading from the muscular layer to the subcutaneous layer (Figure 1C). Positron emission tomography imaging demonstrated increased accumulation of 2‐deoxy‐2‐[18F] fluoro‐D‐glucose in the right pleural thickening (Figure 1D) and right retroperitoneal mass (Figure 1E). There was also evidence of recurrent MALT lymphoma in both orbits (Figure 1F,G). Therefore, a diagnostic surgical biopsy was performed on the thickened right pleural lesion. The final diagnosis was a recurrence of MALT lymphoma. Pathologically, the right pleural mass consisted of lymphoid cells with small to medium mildly constricted nuclei (Figure 2A). Lymphoglandular bodies were also observed (Figure 2A, arrowheads). The plasma cells were focally scattered and partially aggregated (blue arrows). The lymphoid cells had Dutcher bodies, which indicated that the nucleus was packed with immunoglobulin (Figure 2A, white arrows). Therefore, plasmacytoid differentiation was suggested in the tumor cells. On immunohistochemical analysis, the lymphoid cells were predominantly positive for CD20 (Figure 2B), and MUM1 (Multiple Myeloma Oncogene 1), but negative for CD3, CD10, CD5, and cyclin D1. CD138 positive cells were also observed (Figure 2C). The morphological and immunohistochemical findings of the right pleural tumor were similar to those of the initial MALT lymphoma in the right and left orbits. There were few plasma cells, that were in small focal clusters or scattered (Figure 2D–G). Pathological and flow cytometric analysis of bone marrow showed bone marrow involvement of lymphoma. The chromosomal abnormalities of the lymphoma cells were unknown because there was cell‐culture failure in the G‐banding analysis. Therefore, we concluded that the lymphoma of the orbits recurred in the right retroperitoneum. Our final diagnosis was systemic recurrence in the right pleura, right retroperitoneum, and the orbits. The first course was administered as monotherapy with bendamustine alone, without rituximab to avoid the risk of hyperviscosity syndrome. Thereafter, combination therapy of bendamustine and rituximab was administered for the second course. Consequently, the right pleural effusion and patient's symptoms immediately improved. No recurrence has been observed 7 months to date after completion of six courses of treatment with bendamustine and rituximab.

## 3 DISCUSSION

This was a case of MALT lymphoma with metachronous recurrence in the right pleura, retroperitoneum, and orbit. MALT lymphoma is a low‐grade lymphoma of the B‐cell type that originates from lymphoid tissue found in association with mucous membranes and glands and accounts for 7%–8% of non‐Hodgkin's lymphomas.^1^, ^2^ MALT lymphoma often shows localized lesions in extranodal organs such as the stomach, lungs, salivary glands, skin, thyroid, mammary glands, and liver. MALT lymphoma is the most common type of lymphoma in the ocular adnexae.

There are various reports on the usefulness of positron emission tomography (PET) in MALT lymphoma, and sensitivity and specificity vary from organ to organ.^7^, ^8^, ^9^, ^10^, ^11^ In this case, PET scan was useful in examining the extent of the lesions, because PET imaging demonstrated increased accumulation of 2‐deoxy‐2‐[18F] fluoro‐d‐glucose. Though, chromosomal abnormalities of the lymphoma cells were not detectable in this case, the diagnosis of MALT lymphoma is generally aided not only by the clinical picture, histological examination, cytomorphological, and immunophenotypic features, but also by cytogenetic features.^12^

MALT lymphoma comprises 50%–78% of ocular adnexal lymphomas in Western countries, and 80%–90% in Japan and Korea.^4^ Previous reports indicated that the frequency of ocular adnexal lymphoma by site was 59%–75% in the orbit, 25–36% in the conjunctiva, and 2%–5% in the eyelid.^13^, ^14^ The most frequent recurrence sites were similar to the initial sites: 6% progressed at distant sites, 7% at contralateral sites, and 14% at the same sites. Distant invasion have been identified in the Waldeyer's ring, extramedullary mass, stomach, liver, and spleen.^13^ Other reports indicate that ocular adnexal lymphomas (including MALT lymphoma, 43% of all cases) have reportedly spread to multiple sites, the most common being lymph nodes, skin, bone marrow, spleen, and temporalis fossa as the local site.^14^ In these reports, there was no evidence of pleural or retroperitoneal invasion.

Approximately 16% of non‐Hodgkin's lymphoma patients have pleural lesions or develop pleural involvement during the course of their disease.^15^ In particular, diffuse large B‐cell lymphoma (DLBCL) arising in the pleura was commonly observed, but the primary occurrence of MALT lymphoma in the pleura is rare (Table 1).^16^, ^17^, ^18^, ^19^, ^20^, ^21^, ^22^ These patients developed dyspnea as the main symptom. One patient had pleural dissemination of primary lung MALT lymphoma, but the others were diagnosed with MALT lymphoma of pleural origin. Most of them had no associated chronic pleural inflammation, such as pyothorax. In most of the cases, a surgical biopsy of the pleural lesion was performed. Pathology, immunohistochemical studies, and chromosome analysis confirmed the diagnosis of pleural primary MALT lymphoma.^16^, ^17^, ^18^, ^19^, ^20^, ^21^, ^22^

**TABLE 1:** A review of literature on MALT lymphoma arising in the pleura

Case	Age (years)	Sex	Presentation	Associated pulmonary lymphoma	Associated pyothorax	Immunohistochemical studies	Chromosome analysis	Treatment	Clinical outcome
Kodama et al^16^	79	M	Asymptomatic	Yes, pleural dissemination	No	positively with CD20, CD79a and bc1‐2 and stained negatively with CD3, CD21, Cyclin D1, p53 and CD5.	Not reported	Surgical resection	Not reported
Ahmad et al^17^	59	M	Dyspnea, chest pain	No	No	CD43, CD23 and BCl‐2 positive, and CD5 and CD10 negative.	Not reported	Pleurodesis and chemotherapy with chlorambucil	Nonrecurrent after 18 months follow‐up
Ahmad et al^17^	49	M	Dyspnea, cough, weight loss	No	No	CD43, aCD79 and BCl‐2 positive, and CD5, CD10 and CD23 negative.	Not reported	Chemotherapy with chlorambucil and prednisolone	Nonrecurrent after 14 months follow‐up
Hirai et al^18^	72	M	Dyspnea	No	No	Not reported	Not reported	Complete en bloc resection	Not reported
Mitchell et al^19^	47	M	Fever, chest pain	Not reported	Yes (acute)	CD20 positive, and CD5, CD23, CD10, BCL‐6, and cyclin D1 negative	Absence of an Ig heavy chain rearrangement and the 14; 18 translocations.	Pleural decortication	Stable disease
Gomyo et al^20^	67	F	Dyspnea	No	No	CD79a, CD20 and IgM positive, and CD5, CD10, and CD138 negative	t (14;18) (q32; q21)	Chemotherapy with cladribine and rituximab	Complete regression
Kawahara et al^21^	79	M	Back pain	No	No	Bcl2, CD20, CD45RB and CD79a positive, and CD3, CD5, CD10 and cyclin D1 negative	Not reported	Surgical resection	Not reported
Giovanna et al^22^	74	F	Dyspnea, cough	No	No	CD20, kappa light chain positive, and CD5, CD23, CD10, and cyclin‐D1 negative.	Not reported	Pleurodesis and chemotherapy withrituximab	Recurrence after 2 years follow‐up

Abbreviation: MALT, Mucosa associated lymphoid tissue.

Moreover, pleural recurrence resulting from MALT lymphoma is rare. Only one case of pleural recurrence of gastric MALT lymphoma has been reported. A gastric MALT lymphoma patient, who underwent Helicobacter *pylori* eradication, reportedly developed pleural recurrence 1 year later.^5^ Little is known about pleural recurrence secondary to orbital MALT lymphoma in the English literature. There have been few reports on orbital lymphoma with retroperitoneal recurrence. Among the 47 reported cases of orbital lymphomas (including eight MALT lymphomas) treated with radiotherapy, only one patient experienced retroperitoneal recurrence.^6^ Although there is no established follow‐up method for MALT lymphoma, physicians usually suggest a longer follow‐up than for DLBCL (more than 5 years) because MALT lymphoma is a low‐grade lymphoma and late recurrence after 5 years is possible.

In conclusion, the present case focused on the diagnostic challenges in evaluating pleural thickness and the occurrence of a metachronous recurrence from MALT lymphoma. Therefore, pleural effusion should be carefully examined and monitored for recurrence in MALT lymphoma patients. Physicians should be alert to the possibility of recurrence in patients with past history of MALT lymphoma.

## AUTHOR CONTRIBUTIONS

*Conceptualization and methodology*, A.S. and H.I.; *Project administration, visualization, and writing—original draft preparation*, A.S. and H.I.; *Supervision*, T.S., N.T., K.K., K.K., and H.K.; *Investigation and resources*, R.T., R.A., O.Y., K.H., E.N., H.I., Y.M., and A.M.; *Writing —review and editing*, All authors. All authors read and approved the final manuscript.

## CONFLICT OF INTEREST

The authors declare no conflict of interest.

## ETHICS STATEMENT

The article adhered to the principles outlined in the Declaration of Helsinki.

## Data Availability

The data presented in this report are available on request from the corresponding author. The data are not publicly available.
